# Synovial Fluid Immune Cell Landscape in Juvenile Idiopathic Arthritis

**DOI:** 10.1002/eji.70287

**Published:** 2026-10-08

**Authors:** Viola Pitkänen, Bella Lappalainen, Marja Rytkönen‐Nissinen, Terhi Remes‐Pakarinen, Paula Vähäsalo, Milja Möttönen, Minna‐Maija Grönlund, Miika Arvonen, Liisa Kröger, Anna‐Mari Schroderus, Tuure Kinnunen

**Affiliations:** ^1^ Institute of Clinical Medicine University of Eastern Finland Kuopio Finland; ^2^ Department of Children and Adolescents Kuopio University Hospital Kuopio Finland; ^3^ Research Unit of Clinical Medicine University of Oulu Oulu Finland; ^4^ Department of Pediatrics Oulu University Hospital Oulu Finland; ^5^ Department of Pediatrics Turku University Hospital Turku Finland; ^6^ ISLAB Laboratory Centre Kuopio Finland

**Keywords:** autoimmunity, flow cytometry, inflamed joint, juvenile idiopathic arthritis, single‐cell multi‐omics

## Abstract

Juvenile idiopathic arthritis (JIA) is a common inflammatory rheumatic disease of childhood, yet the mechanisms driving immune activation in affected joints remain unclear. In this study, we analyzed synovial fluid (SF) immune cells from patients with JIA using two complementary methodologies, single‐cell multiomics and flow cytometry. SF displayed a markedly altered immune landscape compared with blood. Both myeloid and plasmacytoid dendritic cells were enriched in SF, and SF monocytes exhibited signs of macrophage differentiation. B cells were strikingly rare in SF, and the few B cells present were predominantly CD21^lo^ age‐associated B cells. In contrast, CD56^hi^CD16^−^ NK cells and CD161^−^ γδ T cells were strongly enriched in SF. Among SF CD4^+^ T cells, regulatory T cells, T peripheral helper cells, and CD161^+^ T cells were most abundant, while CD8^+^ T cells exhibited a tissue‐resident memory phenotype with a broad expression of different granzymes. Immune cell frequencies in SF correlated poorly with matched blood samples, and no clear association between SF immune cell composition and JIA subtype or selected clinical parameters was detected. This comprehensive characterization of the SF immune environment provides a foundation for future studies aimed at defining the roles of specific immune cell subsets in JIA pathogenesis.

## Graphical abstract

Synovial fluid immune cells in juvenile idiopathic arthritis were profiled using single‐cell multiomics and flow cytometry. The immune landscape in synovial fluid was markedly distinct from that of blood and was enriched for dendritic cells, CD56^hi^ NK cells, γδ T cells, CD4^+^ T peripheral helper and regulatory T cells, tissue‐resident memory CD8+ T cells, and age‐associated B cells. Immune cell frequencies correlated poorly with clinical parameters in the analyzed patients.

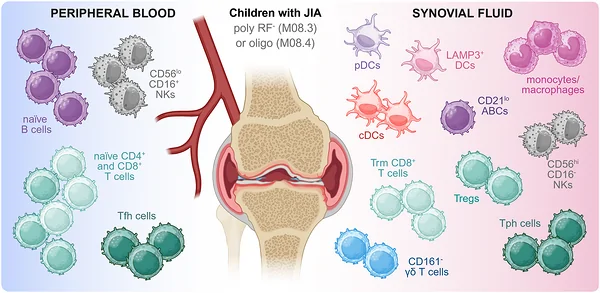

**Abbreviations**
ABC: age‐associated B cell
ANA: antinuclear antibody
DC: dendritic cell
JIA: juvenile idiopathic arthritis
PBMC: peripheral blood mononuclear cell
RA: rheumatoid arthritis
SF: synovial fluid
SFMC: synovial fluid mononuclear cell
ST: synovial tissue
Tfh: T follicular helper cell
Tph: T peripheral helper cell
Treg: T regulatory cell

## 1 Introduction

Juvenile idiopathic arthritis (JIA) is the most common pediatric inflammatory rheumatic disease, affecting more than two million children worldwide [1]. Despite its prevalence, the underlying immune pathology of JIA remains poorly understood, and the response to targeted therapies varies widely among patients [1]. According to the International League of Associations for Rheumatology criteria, JIA is currently classified into seven subtypes [1]. The most common subtypes of JIA are oligoarthritis and rheumatoid factor‐negative polyarthritis, both of which manifest as inflammation of synovial tissue (ST) in one or multiple joints. Together, they account for the vast majority (70%–90%) of all JIA cases [1, 2, 3]. These two subtypes share substantial clinical and genetic similarities, and it has been suggested that they could represent a single disease spectrum, rather than being separate disease entities [4]. Up to 60% of patients with oligo‐ or polyarticular JIA test positive for antinuclear antibodies (ANAs), typically directed against chromatin structures [2]. ANA‐positive patients are more often female, have an earlier age of disease onset, and have an elevated risk of uveitis, indicating that they may constitute a biologically distinct subgroup within the broader JIA disease spectrum [1, 4].

During JIA disease flares, synovial fluid (SF) accumulates in inflamed joints, and it is routinely aspirated before injecting corticosteroids to suppress inflammation. Recent studies suggest that the immune cell composition of SF partially reflects that of ST, particularly for T cells [5, 6]. Therefore, SF could represent an easily accessible sample source to investigate the immune pathogenesis of JIA. Moreover, the SF immune cell profiling could hold biomarker potential for predicting treatment responses, as demonstrated by a recent analysis of ST immune cells in patients with rheumatoid arthritis (RA) [7].

Only a limited number of modern high‐dimensional single‐cell studies have explored the immune cell composition of JIA SF. Aside from one recent study that comprehensively compared immune cells in both SF and ST [6], most single‐cell omics studies have focused primarily on analyzing T cells isolated from SF [8, 9]. In addition to single‐cell omics studies, one recent study used spectral flow cytometry to investigate the immune cell composition of JIA SF [10].

In the current study, we comprehensively compared the immune cell landscape in paired SF and blood samples from patients with oligo‐ or polyarticular JIA using single‐cell omics integrating RNA and protein expression. We further validated and extended these findings in a larger patient cohort using flow cytometry, enabling us to explore how SF immune cell frequencies correlate with clinical parameters and with corresponding blood immune cell frequencies.

## 2 Results

### 2.1 Synovial Fluid and Blood from Patients with JIA Contain All Major Immune Cell Subsets

To broadly compare the immune cell landscape between synovial fluid (SF) and peripheral blood in patients with JIA, we performed single‐cell multi‐omics profiling using the BD Rhapsody Single Cell Analysis System, integrating targeted gene expression (473 genes) and surface protein expression (45 surface proteins). After filtering out low‐quality cells, the dataset comprised 7250 synovial fluid mononuclear cells (SFMCs) from six patients with active synovitis and 2336 peripheral blood mononuclear cells (PBMCs) from two of these patients.

Unsupervised clustering of SFMCs and PBMCs resulted in nine metaclusters, representing canonical immune cell subsets, such as monocytes/macrophages, two subsets of conventional dendritic cells (cDC1 and cDC2), plasmacytoid dendritic cells (pDCs), B cells, CD4^+^ and CD8^+^ T cells, γδ/unconventional T cells (γδ /UCT), and NK cells (Figure 1; Figure S1, Table S1). All these major lineages were detected in both SF and blood, albeit at different reliative abundances.

**FIGURE 1:**
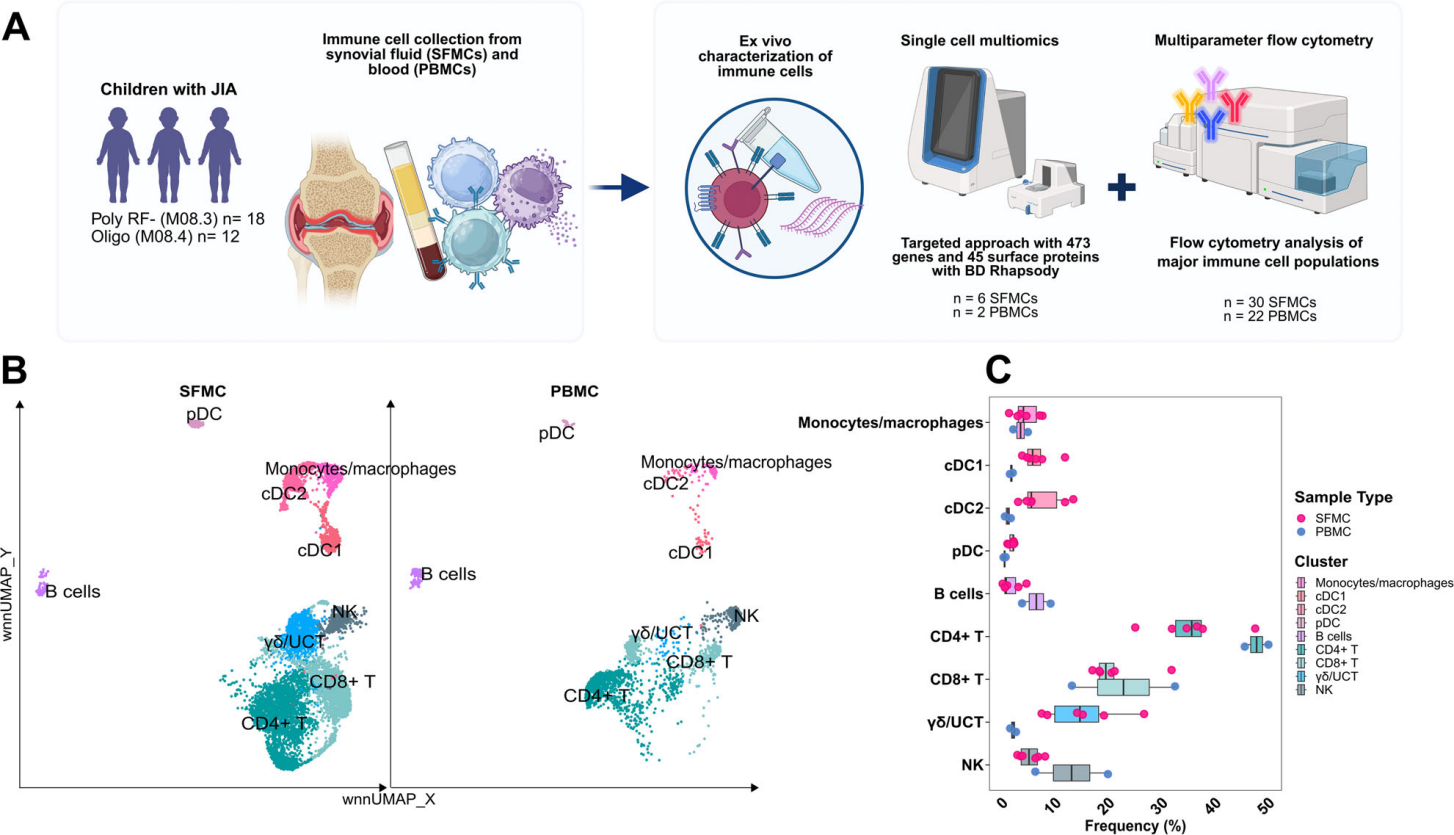
Distinct immune cell landscape in the synovial fluid of children with JIA. Schematic overview of the experimental workflow of the project (A). UMAP projection showing the canonical immune cell landscape of 7250 SFMCs (left) and 2336 PBMCs (right), grouped into immune cell metaclusters. Clustering was performed using Seurat, and metaclusters were annotated manually based on the expression of canonical cell type‐specific surface proteins and genes (B). Frequencies of cells within each metacluster in SFMCs and PBMCs (C). BioRender.com was used to create the schematic overview.

### 2.2 Distinct Monocyte/Macrophage and Dendritic Cell Subsets Are Enriched in Synovial Fluid Compared with Blood

To analyze the identified immune cell subsets at a more granular level, we combined the single‐cell metaclusters containing monocyte/macrophage and dendritic cell subsets and reclustered them into six new clusters (Figures 2A–C; Table S2). One of these clusters (cluster 5) represented residual contaminating CD3^+^ T cells, and it was excluded from further analyses. Cluster 0 consisted of monocytes/macrophages, characterized by the expression of CD14, CD16, and CD11b proteins and transcripts for *SPP1*, *CCL2, S100A9*, and *S100A12*. When comparing cluster 0 between SF and blood using pseudobulk analysis, transcripts characteristic of tissue macrophages associated with chronic inflammation, such as *FN1*, *SPP1*, and *CXCL10* [6], were upregulated in SF, and transcripts enriched in circulating monocytes, such as *CD48*, *SELL*, and *S100A12* [11], were upregulated in blood, respectively (Figure 2D).

**FIGURE 2:**
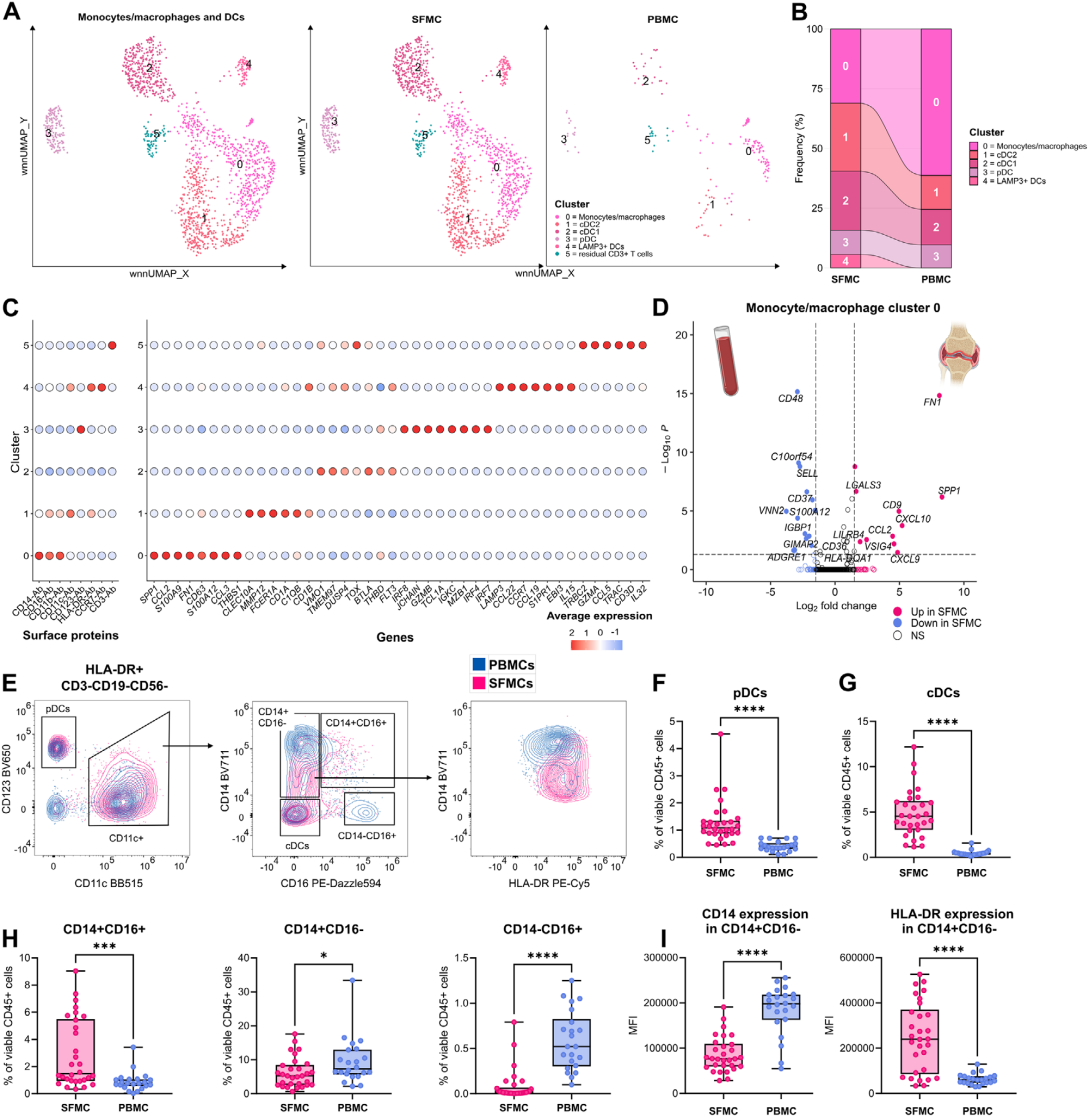
Monocyte/macrophage and dendritic cell subsets in SFMCs and PBMCs from children with JIA. UMAP projection of 1506 SFMCs and 169 PBMCs originating from monocyte/macrophage and dendritic cell (DC) metaclusters, reclustered into five new clusters (left) and shown separately by sample type (right) (A). Proportions of monocyte/macrophage and dendritic cell subsets in SFMCs and PBMCs (B). Expression of selected hallmark surface proteins of monocytes/macrophages and DCs, as well as the top six and selected significantly upregulated genes within each cluster, visualized using a dot plot heatmap (C). Differentially expressed genes in the monocyte/macrophage cluster between SFMCs and PBMCs (D). Representative flow cytometry gating strategy for myeloid and DC subsets, with paired SFMC (pink) and PBMC (blue) samples from a single patient overlaid (E). Frequencies of pDCs (F), cDCs (G), different monocyte/macrophage subsets (H), as well as CD14 and HLA‐DR expression levels (median fluorescence intensity, MFI) in CD14^+^CD16^−^ monocytes/macrophages (I) in SFMCs (pink) and PBMCs (blue). Mann–Whitney *U* test was used for statistical testing. Statistical significance is indicated as *p < 0.05, ***p < 0.001, ****p < 0.0001. BioRender.com was used to create the icons in **D**.

Cluster 3 represented pDCs expressing CD123 and CD45RA at the protein level and transcripts for *IL3RA*, *IRF7*, and *IRF8* [12]. Cluster 2 corresponded to cDC1‐type DCs, defined by *BTLA*, *HLA‐DPA1*, and *THBD* expression, whereas cluster 1 corresponded to cDC2‐type DCs, expressing *CLEC10A* and *FCER1A* [12]. Both cDC clusters were more abundant in SF compared with blood. Finally, cluster 4, detected exclusively in SF, represented a subset of DCs expressing CCR7 and *LAMP3*, together with chemokine and cytokine transcripts including *CCL19*, *CCL22, EBI3, IL15*, and *IL32*.

To validate these findings, we analyzed flow cytometry data from a larger cohort comprising 30 SF and 22 blood samples from JIA patients with active synovitis (Figure S2). Consistent with the single‐cell data, both CD11c^−^CD123^+^ pDCs and CD11c^+^CD14^−^CD16^−^ cDCs were enriched in SF compared with blood (Figure 2E–G). We also subdivided monocytes/macrophages based on CD14 and CD16 expression (Figure 2E,H). Notably, CD14^−^CD16^+^ cells, corresponding to nonclassical monocytes, were virtually absent in SF. Moreover, CD14^+^CD16^−^ cells were modestly decreased, and CD14^+^CD16^+^ cells slightly increased in SF compared with blood. The expression of CD14 within the CD14^+^CD16^−^ monocyte/macrophage subset was decreased, and that of HLA‐DR increased in SF compared with blood (Figure 2I), a finding consistent with a more activated phenotype of these cells in the inflamed joint environment.

### 2.3 The Rare B Cells in Synovial Fluid Are Highly Differentiated and Enriched for CD11c^+^CD21^lo^ Age‐Associated B Cells

Next, B cells in the single‐cell multiomics dataset were reclustered into four subclusters. B cells were present at a much lower frequency in SF compared with blood and displayed distinct phenotypes (Figure 3A–C; Table S3). Blood B cells were predominantly distributed across two naïve B cell clusters (clusters 1 and 2), both expressing IgD but lacking CD27 expression. Of these, cluster 1 corresponded to transitional B cells, characterized by higher expression of IgM, CD38, and *TCL1A*, whereas cluster 2 corresponded to mature naïve B cells [13].

**FIGURE 3:**
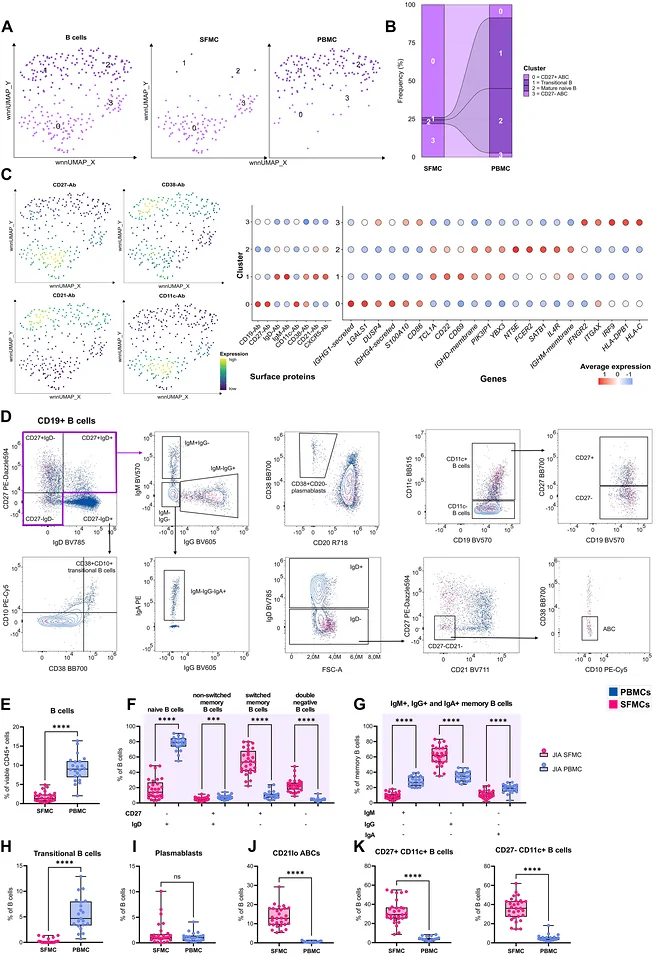
B‐cell subsets in SFMCs and PBMCs from children with JIA. UMAP projection of 119 SFMCs and 151 PBMCs originating from the B‐cell metacluster, reclustered into four new clusters (left), and shown separately by sample type (right) (A). Proportions of B‐cell subsets in SFMCs and PBMCs (B). Expression of CD27, CD38, CD21, and CD11c surface proteins overlaid on the UMAP projection (left), and expression of selected hallmark B‐cell surface proteins and the top six significantly upregulated genes within each cluster visualized using a dot plot heatmap (right) (C). Representative flow cytometry gating strategy for B‐cell subsets, with paired SFMC (pink) and PBMC (blue) samples from a single patient overlaid (D). Frequencies of total CD19^+^ B cells (E), different naïve and memory B‐cell subsets defined by IgD/CD27 expression patterns (F), IgM‐, IgG‐, and IgA‐expressing memory B cells (G), transitional B cells (H), plasmablasts (I), age‐associated CD21^lo^ B cells (ABCs) (J), and CD27^+/−^CD11c^+^ B cells (K) in SFMCs (pink) and PBMCs (blue). Mann–Whitney *U* test was used for statistical testing. Statistical significance is indicated as ***p < 0.001, ****p < 0.0001; ns = nonsignificant.

In contrast to blood, SF B cells were enriched in clusters 0 and 3, both lacking the expression of IgD, IgM, and CD21 but expressing CD11c/*ITGAX*, consistent with the phenotype assigned to age‐associated B cells (ABCs) [14]. Of these, cluster 0 expressed higher levels of CD27 and transcripts for IgG heavy chains (*IGHG1* and *IGHG2*).

We validated these findings again in the larger flow cytometry dataset (Figure S3). In line with the single‐cell omics data, the mean B‐cell frequency was approximately seven‐fold lower in SF than in blood (1.3% vs. 9.0% of total CD45^+^ cells) (Figure 3D,E). Classifying B cells into four subsets based on the expression of IgD and CD27 (Figure 3D), we observed that IgD^−^CD27^+^ switched memory and IgD^−^CD27^−^ double‐negative B cells [13] were substantially more abundant in SF, whereas IgD^+^CD27^−^ naïve and IgD^+^CD27^+^ nonswitched memory B cells were correspondingly reduced (Figure 3F). Furthermore, the frequency of IgG‐class memory B cells was higher in SF compared with blood, whereas the frequencies of IgM‐ and IgA‐class memory B cells were lower (Figure 3G).

Notably, CD38^+^CD10^+^ transitional naïve B cells were virtually absent in SF (Figure 3H), whereas CD38^+^CD20^lo^ plasmablasts were detected at comparable frequencies in both blood and SF (Figure 3I). Finally, using the definition of IgD^−^CD27^−^CD21^lo^CD38^−^CD10^−^ for ABCs [15, 16, 17], we confirmed that they were markedly enriched in SF relative to blood (Figure 3J). Consistent with this, the frequency of CD11c^+^ B cells, both within the CD27^+^ and CD27^−^ compartments, was higher in SF than in blood (Figure 3K).

### 2.4 CD56^hi^ NK Cells and CD161‐Negative γδ T Cells Are Enriched in SF Compared with Blood

Finally, we reclustered the T‐cell and NK‐cell single‐cell metaclusters into 13 new clusters (Figure 4A–C; Table S4). Among these, clusters 7 and 9 represented NK cells, with cluster 7 predominantly enriched in SF and cluster 9 in blood. Both clusters expressed canonical NK‐cell transcripts, including *TYROBP*, *FCER1G*, and *KLRF1* [18]. Cluster 7 was further characterized by higher expression of CD56 and *KLRC1*, whereas cluster 9 showed higher expression of CD16 and the transcripts *FCGR3A*, PRF1, *CCL4*, and *CCL3*, corresponding to CD56^hi^CD16^−^ and CD56^lo^CD16^+^ NK‐cell subsets, respectively [19]. These findings were again validated in the larger flow cytometry dataset (Figure S2): both CD56^hi^CD16^−^ and CD56^hi^CD16^+^ NK‐cell subsets were more abundant in SF than in blood, whereas CD56^lo^CD16^+^ were virtually absent in SF (Figure 4D,E).

**FIGURE 4:**
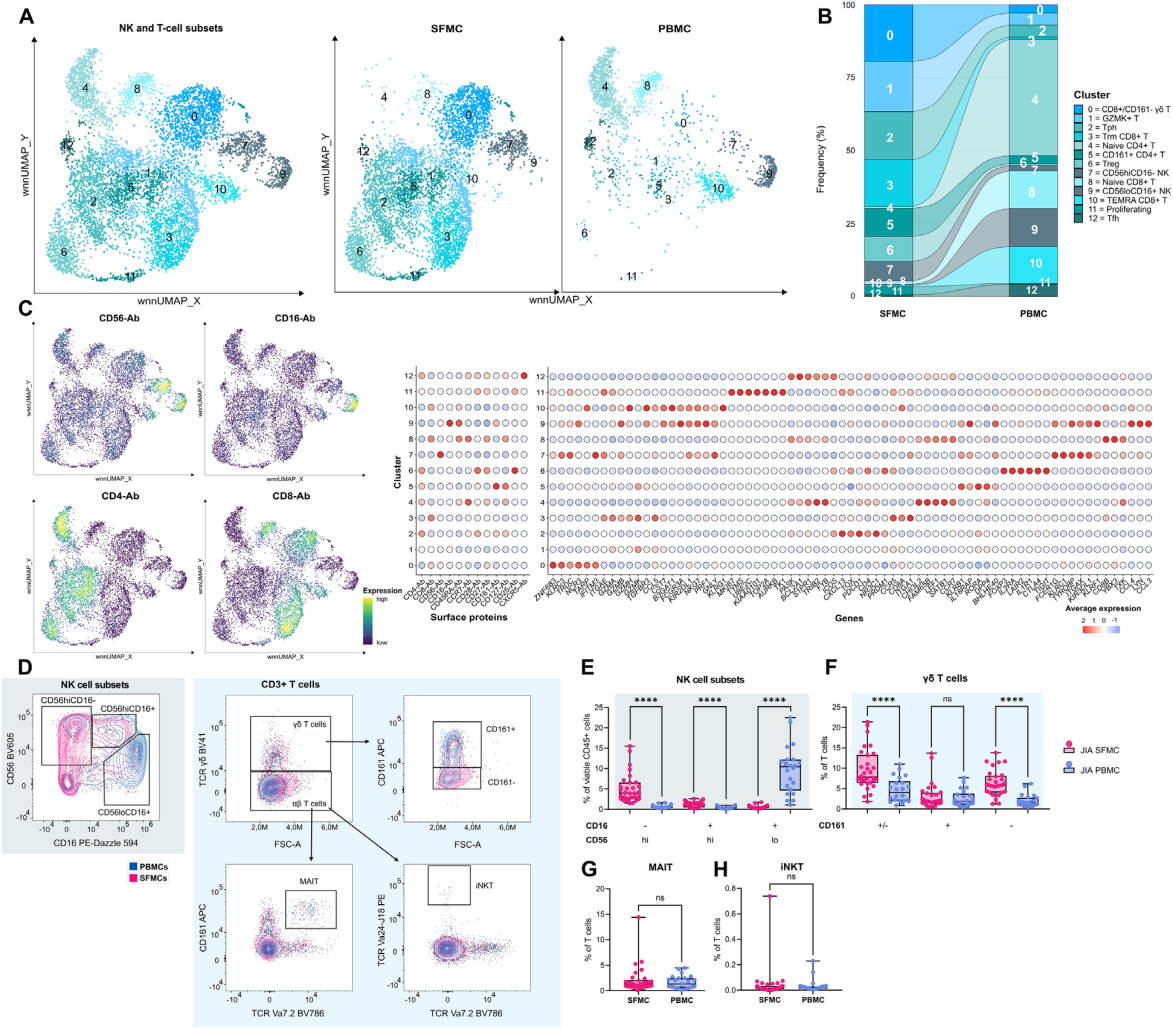
T‐cell and NK‐cell subsets in SFMCs and PBMCs from children with JIA. UMAP projection of 5,625 SFMCs and 2,016 PBMCs corresponding to T‐cell and NK‐cell metaclusters, reclustered into 13 new clusters (left) and shown separately by sample type (right) (A). Proportions of T‐cell and NK‐cell subsets in SMFCs and PBMCs (B). Expression of CD56, CD16, CD4, and CD8 surface proteins overlaid on the UMAP projection, and expression of selected hallmark T‐ and NK‐cell surface proteins together with the top six and selected significantly upregulated genes within each cluster visualized using a dot plot heatmap (C). Representative flow cytometry gating strategy for NK‐cell subsets (CD56^hi/lo^CD16^+/−^) and unconventional T‐cell subsets: CD161^+/−^ γδ T cells, MAIT cells (TCRVα7.2^+^CD161^+^), and iNKT cells (TCR Vα24‐Jα18^+^), with paired SFMC (pink) and PBMC (blue) samples from a single patient overlaid (D). Frequencies of different NK‐cell subsets (E), γδ T‐cell subsets (F), MAIT cells (G), and iNKT cells (H) in SFMCs (pink) and PBMCs (blue). Mann–Whitney *U* test was used for statistical testing. Statistical significance is indicated as ****p < 0.0001; ns = nonsignificant.

Cluster 0 corresponded to CD161^−^ γδ T cells, characterized by the expression of *TRDC* and *TARP*. Notably, these CD161^−^ γδ T cells appeared enriched in SF compared with blood, a result confirmed by flow cytometry (Figure 4D,F; Figure S4). Finally, we assessed the frequencies of two additional innate‐like T‐cell subsets, MAIT cells and iNKT cells, by flow cytometry (Figure 4D), as their low abundance likely precluded reliable detection by the single‐cell multiomics approach. However, their frequencies did not to differ between SF and blood (Figure 4G,H).

### 2.5 CD4^+^ T Cells in Synovial Fluid Are Enriched for Regulatory and Peripheral Helper T Cells, Whereas CD8^+^ T Cells Display a Tissue‐Resident Effector Memory Phenotype

The remaining ten clusters corresponded to conventional CD4^+^ and CD8^+^ T cells. Cluster 11 contained highly proliferating T cells, characterized by strong expression of cell‐cycle‐associated genes such as *TYMS*, *UBE2C*, and *MKI67*, and was highly enriched in SF compared with blood (Figure 4A–C).

Among the four CD4^+^ T cell clusters, cluster 4 represented naïve CD4^+^ T cells, defined by high expression of CD45RA, CCR7, *LEF1*, *SATB1*, and *CCR7*. Cluster 5 corresponded to CD161^+^ memory CD4^+^ T cells, expressing CD161, CD127, *KLRB1, CXCR3*, and *RORA*. Cluster 6 represented regulatory T cells (Tregs), marked by high expression of CD25, *FOXP3, TIGIT*, and *CTLA4*. Cluster 2 matched the profile of T peripheral helper (Tph) cells, with high expression of PD‐1, ICOS, BTLA, *CXCL13*, and *TOX*, but lacking CXCR5 [20]. In turn, cluster 12 corresponded to T follicular helper (Tfh) cells, expressing CXCR5, BTLA, and *ICOS*. Of these clusters, CD161^+^ memory CD4^+^ T cells, Tregs, and Tph cells were strongly enriched in SF, whereas naïve CD4^+^ T cells and Tfh cells were largely absent.

Three CD8^+^ T cell clusters were also identified. Of these, cluster 8 represented naïve CD8^+^ T cells, with high expression of CD45RA, CCR7, CD27, and *LEF1* and *CCR7*. Cluster 10 corresponded to CD8^+^ TEMRA cells, with high expression of CD45RA, CX3CR1 and *B3GAT1*, *FCGR3A*, *KIR2DL1*, *NKG7*, *PRF1*, and *GNLY*, but low expression of *CCR7* and CD27. Both naïve and TEMRA CD8^+^ T cells were strongly enriched in blood relative to SF. In contrast, cluster 3 was heavily enriched in SF and displayed a transcriptomic profile consistent with tissue‐resident memory CD8^+^ T cells, including high expression of PD‐1, CD69, *ITGAE*, *CCL5*, and transcripts encoding multiple granzymes (*GZMA, GZMB, GZMH, GZMK)* [21]. The closely related cluster 1 also expressed high levels of *GZMA, GZMK*, and *CCL5*, but contained both CD4^+^ and CD8^+^ T cells.

Flow cytometry analyses in the larger sample set confirmed these observations (Figure 5; Figure S4). Overall, the proportion of CD4^+^ T cells was reduced, whereas that of CD8^+^ T cells was increased, in SF compared with blood (Figure 5A,B). Memory CD4^+^ and CD8^+^ T‐cell subsets [13], apart from the rare CD8^+^CD27^−^ TEMRA population, predominated in SF compared with blood (Figure 5A,C,D). Furthermore, CD4^+^CD25^+^CD127^lo^FOXP3^+^ Tregs were markedly enriched in SF (Figure 5A,E). Finally, CXCR5^+^ Tfh cells were almost completely absent from SF, while CXCR5^−^PD‐1^hi^ T cells, corresponding to the phenotypic definition of Tph cells [20], were strongly enriched (Figure 5A,F).

**FIGURE 5:**
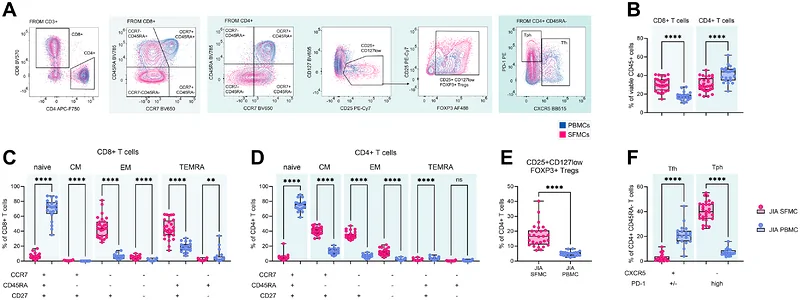
CD8^+^ and CD4^+^ T‐cell subsets in SFMCs and PBMCs from children with JIA. Representative flow cytometry gating strategy for CD8^+^ and CD4^+^ T‐cell subsets, with paired SFMC (pink) and PBMC (blue) samples from a single patient overlaid (A). Frequencies of total CD8^+^ and CD4^+^ T cells (B), naïve and memory subsets of CD8^+^ T cells (C) and CD4^+^ T cells (D) defined by CD45RA/CCR7/CD27 expression patterns, total CD4^+^ Tregs (E), and CD4^+^ CXCR5^+^ Tfh and CXCR5^−^PD‐1^hi^ Tph cells (F) in SFMCs (pink) and PBMCs (blue). Mann–Whitney *U* test was used for statistical testing. Statistical significance is indicated as *p < 0.05, **p < 0.01, ***p < 0.001, ****p < 0.0001; ns = nonsignificant.

### 2.6 Poor Correlation of Synovial Fluid Immune Cell Frequencies With Clinical Parameters and Blood Immune Cell Frequencies

Finally, we performed an exploratory analysis to determine whether SF immune cell frequencies, analyzed by flow cytometry in the 30 JIA patients, were associated with clinical characteristics of the patients. These included JIA category (oligoarthritis or rheumatoid factor‐negative polyarthritis), antinuclear antibody positivity [2], age, sex, presence of uveitis, erythrocyte sedimentation rate (ESR) and C‐reactive protein (CRP) values, the Juvenile Arthritis Disease Activity Score 10 (JADAS10), and whether joint aspiration was performed at the time of first clinical joint inflammation or during a subsequent relapse. However, no clear associations were identified between any SF immune cell subset and these clinical parameters (Table S5; Figures S5–S7).

We also examined correlations between immune cell frequencies in SF and blood from the 22 paired samples analyzed. Interestingly, the frequencies of rare CD56^hi^CD16^+^ NK cells and iNKT cells, as well as those of CD27^−^CD8^+^ and CD27^−^CD4^+^ TEMRA cells, showed significant correlations between SF and blood. In contrast, the frequencies of monocyte/macrophage and DC subsets, and the majority of B‐cell, CD4^+^ T‐cell, and CD8^+^ T‐cell subsets, did not correlate between SF and blood (Figure 6).

**FIGURE 6:**
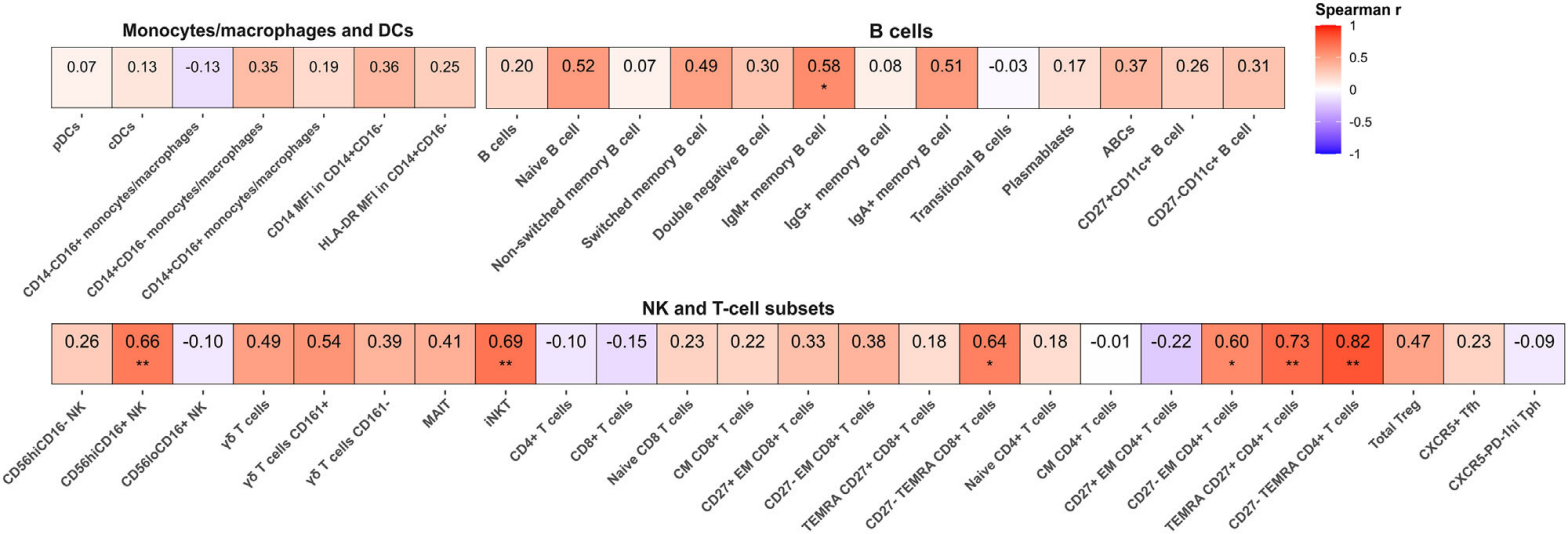
Correlation of immune cell frequencies between SF and blood. Spearman's correlation was used to generate the correlation matrix. Numbers within each square indicate the calculated Spearman's correlation coefficients, and the color scale represents positive correlations in shades of red and negative correlations in shades of blue. Statistical significance is indicated as *p < 0.05, **p < 0.01; *p*‐values are FDR‐corrected.

## 3 Discussion

In this study, we comprehensively profiled the immune cell composition of SF in comparison with peripheral blood from patients with JIA. We used two complementary single‐cell methodologies: targeted single‐cell multiomics integrating protein and mRNA expression and flow cytometry, allowing for both high‐dimensional exploratory analysis in a limited set of patients, as well as validation of the major findings in a larger cohort. We identified several differences in immune cell frequencies and phenotypes between SF and blood. However, the immune landscape of SF did not show clear associations with the clinical parameters used to define JIA subtypes or disease activity.

The myeloid compartment of both SF and blood contained monocytes/macrophages and multiple types of cDCs. Consistent with earlier findings [6, 10], cDCs were more abundant in SF. Although monocytes/macrophages clustered together in our single‐cell multiomics analysis, those in SF expressed a gene signature characteristic of SPP1^+^ macrophages, the dominant myeloid population previously described in JIA SF and ST samples [6]. In line with earlier reports [10, 22], CD14^−^CD16^+^ inflammatory monocytes were virtually absent in SF, whereas CD14^+^CD16^+^ monocytes/macrophages were slightly enriched. Moreover, CD14^+^ monocytes/macrophages in SF exhibited higher HLA class II expression than their blood counterparts, consistent with their enhanced capacity to activate T cells in vitro [22].

Both cDC1 and cDC2 subsets were enriched in SF compared with blood, again in accordance with previous observations [6]. Single‐cell multiomics data further identified a third DC population characterized by high expression of CCR7 and *LAMP3* along with various chemokines and cytokines, exclusively present in SF. Such CCR7^+^LAMP3^+^ DCs have been proposed to represent a specific activation state of DCs linked to immunogenic, regulatory, and migratory gene programs shaped by the microenvironment [23]. Importantly, these cells have also been detected in the ST of both JIA and RA patients with active synovitis [6, 24, 25], where they appear to localize to ectopic lymphoid structures and may contribute to the activation of Tph cells within these structures. pDCs were also present in SF at a slightly higher frequency compared with blood.

The most prominent feature of B cells was their markedly reduced frequency in SF compared with blood, a finding consistent with other studies [6, 10]. Moreover, the SF B‐cell compartment was almost completely devoid of naïve B cells and instead dominated by highly differentiated memory B cells, primarily producing IgG. Importantly, a substantial proportion of SF B cells expressed CD11c (*ITGAX*) and lacked CD21, features characteristic of age‐associated B cells (ABCs). ABCs are linked to chronic inflammation and autoimmunity [14, 15, 16] and have also previously been reported to accumulate in JIA SF [6, 17, 26]. Although ABCs have classically been defined as CD27^−^ [15, 16], nearly half of the CD11c^+^ B cells in SF expressed CD27, indicating additional heterogeneity within this subset, as suggested previously [14, 17, 27].

NK cell subsets demonstrated a clear dichotomy between SF and blood. CD56^lo^CD16^+^ NK cells, the predominant subset in blood, were virtually absent in SF, while CD56^hi^CD16^−^ NK cells were strongly enriched in SF, consistent with prior studies [6, 10]. These two NK cell subsets are thought to have distinct biological roles, with CD56^lo^CD16^+^ NKs being strongly cytotoxic, whereas CD56^hi^CD16^−^ NKs are considered to play an immunomodulatory role [28]. Among innate‐like T cells, γδ T cells [9], and particularly a CD161^−^ subset, were enriched in SF compared with blood. Previous studies have demonstrated that the expression of CD161, a C‐type lectin‐like receptor, correlates with the capacity of T cells, including CD4^+^, CD8^+^, and γδ T cells, to produce IL‐17A [29, 30]. Therefore, the lack of CD161 expression may reflect functional specialization of γδ T cells within the joint microenvironment. The other major innate‐like T cell populations, MAIT and iNKT cells, did not differ in frequency between SF and blood.

Finally, in line with previous studies [6, 10], SF contained increased proportions of CD8^+^ T cells and decreased proportions of CD4^+^ T cells relative to blood, and most SF T cells displayed central or effector memory phenotypes. Tregs were also strongly enriched in SF compared with blood [6, 9, 10]. Tph cells, characterized by high PD‐1 and CXCL13 expression, formed another prominent CD4^+^ T‐cell subset enriched in inflamed SF [6, 8, 9, 31]. These cells likely support B‐cell responses in inflamed tissues and could play an important role in maintaining ectopic lymphoid structures, which are observed in JIA ST [25, 32]. In contrast, CXCR5^+^ Tfh cells, key regulators of B‐cell responses in lymphoid organs, were largely absent from SF. Another CD4^+^ T‐cell subset enriched in SF consisted of CD161^+^ T cells expressing *RORA* and *CXCR3* [6]. They appear to phenotypically align with so‐called Th17/Th1 or nonclassical Th1 cells, which are thought to arise from classical Th17 cells and have been previously identified in JIA SF [33, 34].

CD8^+^ T cells in SF exhibited a phenotype consistent with tissue‐resident memory cells, including high *ITGAE* (CD103) and *CD69* expression [6]. These cells also expressed elevated levels of transcripts for different granzymes (*GZMA*, *GZMB*, *GZMH*, and *GZMK*), but low levels of transcripts for cytotoxic effector molecules (*GNLY* and *PRF1*). These cells likely correspond to highly inflammatory CD8^+^ T cells previously reported to be enriched in SF and ST of patients with RA and JIA [6, 21, 25]. Of note, some SF CD4^+^ T cells also expressed *GZMA* and *GZMK*. Moreover, the frequency of proliferating CD4^+^ and CD8^+^ T cells was clearly higher in SF compared with blood.

Blood samples can be easily collected from JIA patients for diagnostic and research purposes. SF is also routinely aspirated from inflamed joints of JIA patients. However, a critical question for exploratory immune analyses and biomarker development is how well blood and SF reflect the immune cell composition of ST, which is far more challenging to sample. A few recent studies have compared the immune cell composition between SF and ST [5, 6]. CD4^+^ and CD8^+^ T‐cell subsets appear to be present at broadly similar frequencies in SF and ST and demonstrate a high degree of clonal sharing [5, 6]. However, certain B‐cell subsets, particularly plasma cells, are considerably more abundant in ST, and the frequencies of mDCs, pDCs, NK cells, and γδ T cells appear to be higher in SF [6]. Transcriptomic differences between matched SF and ST immune cells have also been reported [6]. In the current study, SF frequencies of most immune cell subsets showed poor correlation with their blood frequencies, consistent with a previous comparison between blood and ST [6]. The major exceptions were the frequencies of CD56^hi^CD16^+^ NK cells, iNKT cells, as well as CD4^+^ and CD8^+^ TEMRA cells. Based on this, these cell types in SF can be speculated to be bystander populations migrating directly from blood rather than deriving primarily from ST. Taken together, SF appears to be a specimen type distinct from both ST and blood, with some cell types likely migrating to SF from circulation and others from ST. In line with this interpretation, a recent spatial transcriptomics study demonstrated that at least SPP1^+^ macrophages, LAMP3^+^ DCs, and cDCs reside in the ST lining layer, from where they can be sheared into SF, potentially explaining their high frequency in SF [6].

We did not identify major associations between SF immune cell composition and JIA subtype (oligo‐ or polyarticular), ANA status, or clinical parameters of disease activity. These findings are consistent with two other recent studies demonstrating minimal immunological differences between SF from patients with oligoarticular and polyarticular JIA [6, 10]. Collectively, these results support the emerging view that these JIA subtypes may represent a single disease spectrum rather than distinct pathogenic entities [4]. Moreover, previous studies have demonstrated that the ST immune landscape in patients with JIA closely resembles that of patients with RA [6, 7, 27]. In a direct comparison, JIA ST appeared to contain slightly more T and B cells and fewer NK and myeloid cells compared with RA ST [6]. Taken together, both our current work and the previous studies suggest that the immune landscape of inflamed ST and SF may be broadly similar across different forms of inflammatory arthritis, potentially limiting the biomarker potential of SF immunophenotyping. Nonetheless, focused analyses of specific rare or functionally specialized immune cell subsets, potentially missed by broad immune landscape studies, may yet reveal clinically meaningful differences. For example, modest increases in SF Tph cells [31] or CD21^lo^ ABCs [17] have been reported in ANA‐positive JIA patients compared with ANA‐negative patients.

A key strength of the current study is the relatively large cohort of SF samples analyzed (*n* = 30), which exceeds the sample sizes of previous SF immune‐profiling studies using either flow cytometry (*n* = 18) [10] or single‐cell transcriptomics (*n* = 9) [6]. Moreover, all samples were derived from patients not receiving immunomodulatory medications, thereby minimizing potential treatment‐related effects on the immune composition of SF. Our combined use of single‐cell multiomics and flow cytometry leveraged the advantages of each approach. Single‐cell multiomics enables broad and largely unbiased characterization of gene and protein expression, but is currently limited by the number of clinical samples that can be feasibly analyzed. In contrast, flow cytometry enables validation in substantially larger cohorts, using a more limited but well‐established set of protein markers supported by decades of experimental work.

Taken together, our analyses provide a comprehensive immune landscape of JIA SF and serve as a foundation for more detailed studies aimed at elucidating the functions and interactions of specific SF immune cell subsets. Such efforts will be essential for increasing our understanding of JIA immunopathogenesis and for guiding future biomarker discovery.

### 3.1 Data Limitations and Perspectives

Our study also has limitations. First, our analyses were limited to CD45^+^ mononuclear cells from SF and blood, excluding neutrophils, which may play an important role in JIA immunopathogenesis [35, 36], from the analyses. Second, although our single‐cell omics panel covered a broad range of genes and proteins, it remained a targeted approach. Combined with the limited number of single cells that could be profiled per sample, this may have prevented us from detecting rare immune subsets or subtle phenotypic states that could still be relevant to JIA pathogenesis. Consequently, larger‐scale single‐cell omics studies will likely be required to identify minor changes in SF immune cell composition linked to JIA subtype or disease activity, particularly for immune cell subsets such as Tph cells or LAMP3^+^ DCs that are difficult to resolve through protein marker expression alone.

Several of the immune populations identified here, including Tph cells, CD21^lo^ B cells, SPP1^+^ macrophages and LAMP3+ DCs, have been shown to be enriched in inflamed ST and SF in both JIA and RA [6, 20, 24, 25, 27]. Although our study was not designed to define causal mechanisms, the enrichment of these populations supports a model in which activated myeloid cells and dendritic cells could provide signals driving the recruitment and retention of Tph and other tissue‐resident T cells, while Tph cells may support the differentiation and persistence of autoreactive B‐cell populations. Future therapeutic strategies targeting these cellular interactions may therefore represent a promising approach for the treatment of JIA.

## 4 Materials and Methods

### 4.1 Study Cohort

The study cohort comprised paired SF (*n* = 30) and blood (*n* = 22) samples collected between 2022 and 2024 from pediatric patients with JIA, recruited at the Kuopio and Turku University Hospitals. All patients were diagnosed with either oligoarthritis (M08.4) or rheumatoid factor‐negative polyarthritis (M08.3), according to the International League of Associations for Rheumatology criteria [1]. The patients had active synovitis, either the first clinical joint inflammation at diagnosis or subsequent disease relapse, and were either treatment‐naïve or had not used immunomodulatory medications for at least 2 months before sampling. A more detailed description of the study cohort demographics and clinical characteristics is provided in Table S6.

### 4.2 Mononuclear Cell Isolation

SF was collected at the time of therapeutic joint aspiration and paired peripheral blood was collected by venipuncture into lithium heparin collection tubes. SF samples were treated with hyaluronidase (10 units/mL, Sigma‐Aldrich) for 30 min at 37°C and subsequently filtered using a 70 µm nylon strainer (Falcon). Mononuclear cells from SF (SFMC) and peripheral blood (PBMC) were isolated by Ficoll density gradient centrifugation and cryopreserved in 10% DMSO in FBS.

### 4.3 Single‐Cell Capture

Single‐cell multiomics analyses were performed using the BD Rhapsody Single‐Cell Analysis (BD Bioscience) platform on samples from six JIA patients (Table S7). Thawed SFMCs (*n* = 6) and PBMCs (*n* = 2) were labeled with CD45 APC‐F750 (BioLegend) and the Single Cell Sample Multiplexing Kit (BD Biosciences) for 20 min at RT. Next, viability staining was performed using 7‐AAD (BioLegend) for 20 min on ice. 30,000 viable (CD45^+^7‐AAD^−^) immune cells from each sample were sorted using the Sony MA900 cell sorter (Sony Biotechnology) and pooled into a single tube. Fc receptors were blocked using Human Fc Block (BD Biosciences) for 10 min at RT, followed by labelling with BD Abseq oligonucleotide‐conjugated monoclonal antibodies (Immune Discovery Panel and 15 add‐in AbSeq Ab‐Oligos, BD Biosciences; Table S8) for 60 min on ice. The cells were subsequently captured using the BD Rhapsody Express System (BD Biosciences), according to the manufacturer's instructions.

### 4.4 Library Preparation

Libraries were prepared according to the manufacturer's protocol (mRNA Targeted, Sample Tag, and BD AbSeq Library Preparation with the BD Rhapsody Targeted mRNA and AbSeq Amplification Kit, Doc ID: 214508 Rev. 3.0 23‐21335‐00) for mRNA expression of 473 genes (targeted approach using 399 genes in the Human Immune Response panel and 76 supplemental genes, BD Biosciences; Table S9), for expression of 45 surface proteins (AbSeq Immune Discovery Panel and 15 additional AbSeq Ab‐Oligos, BD Biosciences; Table S8) and for Sample Tags (Single Cell Sample Multiplexing Kit, BD Biosciences) to perform multiplexing. Essentially, the library preparation was performed as previously described [37]. The final libraries were pooled at 2 nM with a 20% PhiX spike‐in. Sequencing was performed using an SP flow cell, 100‐cycle kit, read length 60 bp + 42 bp (read 1 and read 2, respectively) using a NovaSeq 6000 (Illumina).

### 4.5 Single‐Cell Multiomics Analyses

FASTA reference files and FASTQ sequencing reads were uploaded to SevenBridges (https://www.sevenbridges.com, Velsera). The sequence reads were aligned to the reference genome hg38 using the Human Immune Response Panel (BD Biosciences) and Immune Discovery AbSeq panel (BD Biosciences) FASTA files in SevenBridges using the BD Rhapsody Sequence Analysis Pipeline (revision 17). In total, 10,780 single cells were obtained. Cells without multiplexing information, cells labeled as multiplets, and cells with <15 detected genes were filtered out. Genes expressed in <5 cells were also removed from the dataset. After these filtering steps, 9586 single cells (2336 PBMCs and 7250 SFMCs), 423 genes, and 45 surface proteins were subjected to downstream analyses using Seurat 4.3.0 [38] in R 4.2.3 [39]. Briefly, RNA parameters were normalized using the LogNormalize function, and AbSeq protein expression data were normalized using CLR normalization via the NormalizeData function. FindMultiModalNeighbors function was used to construct a weighted nearest neighbor (WNN) graph. Cell clusters in metacluster and reclustering analyses were identified using the WNN graph by running the FindClusters function. runUMAP function was used to create a UMAP dimensional reduction of the data.

Initial clustering yielded 13 clusters (Figure S1), and well‐known cell type‐specific marker genes expressed by each cluster were used to annotate metaclusters consisting of monocytes/macrophages, DCs (cDC1, cDC2, pDCs), B cells, as well as CD4^+^ T, CD8^+^ T, γδ T/UCT, and NK cells. To characterize each metacluster in more detail, monocyte and DC subsets were selected from the data and reclustered, yielding five new clusters; B cells were reclustered, yielding four clusters; and CD4^+^ T, CD8^+^ T, γδ T/UCT cells, and NK subsets were reclustered, yielding 13 clusters. FindAllMarkers was used for each set of reclustered metaclusters to examine hallmark gene and protein expression in each cluster. Additionally, for B cell clusters, FindMarkers was used between cluster 3 (CD27^−^ ABC) and cluster 2 (mature naive B) to compare gene and protein expression. The log2 fold‐change threshold used in the analyses was 0.25, and a Benjamini‐Hochberg adjusted *p*‐value ≤ 0.05 was considered statistically significant. Wilcoxon rank‐sum test was used as a statistical test.

Pseudobulk analysis for comparing gene expression of the monocyte/macrophage cluster between SFMCs and PBMCs was performed using Seurat 5.1.0 [40]. In brief, the AggregateExpression function was used to create a pseudobulk profile for SFMC and PBMC samples for the cluster, and the FindMarkers function was utilized for differential gene expression analysis using the DESeq2 method.

The following R packages were used for visualizing the data: EnhancedVolcano (version 1.20.0), ggalluvial (version 0.12.5), Nebulosa (version 1.8.0), and ggplot2 (version 4.0.0).

### 4.6 Flow Cytometry Analyses

Viability staining for thawed SFMCs and PBMCs was performed using Zombie Aqua dye (BioLegend) according to the manufacturer's instructions. Staining for surface markers (Table S10) was performed by incubating the cells with fluorochrome‐conjugated antibodies for 20 min at RT in the presence of Brilliant Stain Buffer Plus (BD Biosciences). For the analysis of FOXP3 expression, samples were fixed and permeabilized for 60 min using the Foxp3/Transcription Factor Staining Buffer set (eBioscience) before a 30‐min intracellular staining step at RT. The immunostained samples were acquired with a four‐laser NovoCyte Quanteon flow cytometer (Agilent). The flow cytometry data were analyzed using FlowJo software (FlowJo, version 10.10.0). All flow cytometry analyses were performed in accordance with the “Guidelines for the use of flow cytometry and cell sorting in immunological studies” [13].

### 4.7 Statistics

Statistical analyses were performed in Prism software (GraphPad, version 10.4.1). Mann–Whitney *U* test was used when comparing two groups: cell frequencies between SFMCs and PBMCs, and cell frequencies in SFMCs between groups divided according to dichotomous clinical parameters: ANA status, sex, JIA subtype, or presence of uveitis. Spearman's correlation analysis was used to examine correlations of cell frequencies between SFMCs and PBMCs, and correlations between cell frequencies in SFMCs and continuous clinical parameters: juvenile arthritis disease activity score 10 (JADAS10), erythrocyte sedimentation rate (ESR), C‐reactive protein (CRP) level, and age at diagnosis. Benjamini‐Hochberg false discovery rate (FDR) correction was applied as indicated. A *p*‐value <0.05 was considered statistically significant.

## Author Contributions

V.P., B.L., M.R.‐N., and A.‐M.S. performed the experiments; T.R.‐P., P.V., M.M., M.‐M.G., M.A., and L.K. provided the clinical samples; V.P., A.‐M.S., and T.K. analyzed the data and wrote the manuscript.

## Funding

The study was financially supported by the Foundation for Pediatric Research, Päivikki and Sakari Sohlberg Foundation, and the ImmuDocs Doctoral Education Pilot.

## Ethics Approval and Patient Consent Statements

The study was approved by the local ethics committees of the Kuopio and Turku University Hospitals. All study participants and/or their legal guardians provided written informed consent, in accordance with the Declaration of Helsinki.

## Conflicts of Interest

The authors declare no conflicts of interest.

## Supporting information




**Supporting File 1**: eji70287‐sup‐0001‐SuppMat.pdf.

**Supporting File 2**: eji70287‐sup‐0002‐Tables.xlsx.

## Data Availability

Single‐cell multiomics data generated in this study are deposited at ImmPort (https://www.immport.org, accession number SDY3464). Flow cytometry data are available from the corresponding author upon reasonable request.
