# Supplementary material for: Synovial Fluid Immune Cell Landscape in Juvenile Idiopathic Arthritis
Source: Eur J Immunol. 2026 Oct 8;56(10):e70287. doi: 10.1002/eji.70287 (PMC13647478; doi:10.1002/eji.70287)
Supplement: Supplementary file 1 [file EJI-56-e70287-s001.pdf]

SUPPLEMENTAL MATERIAL

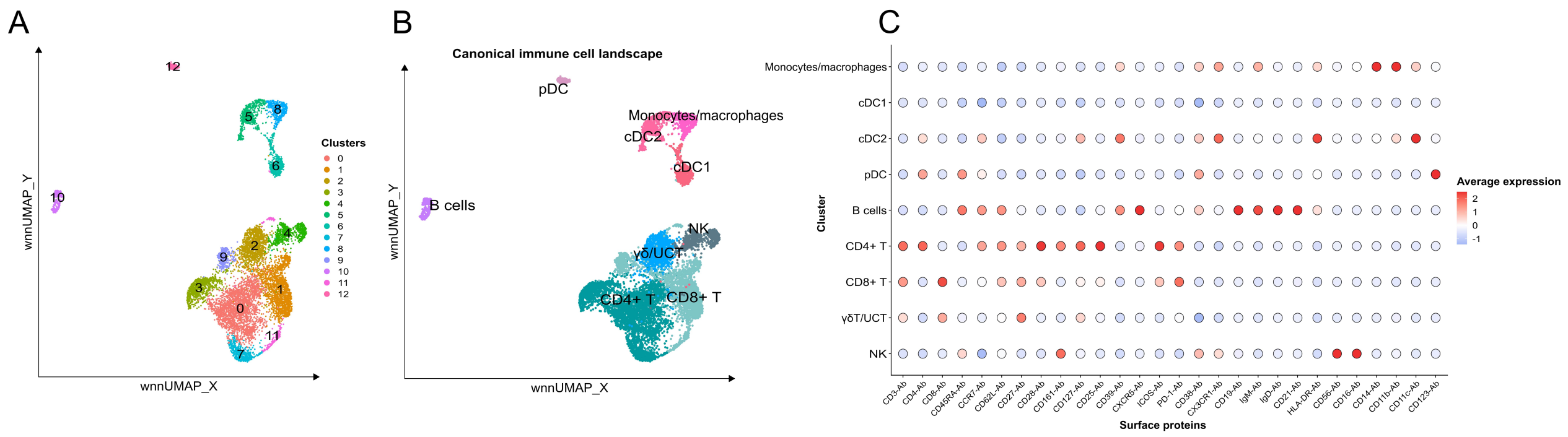

Supplemental Figure 1. Distinct immune cell landscape in the synovial fluid of children with JIA. Initial clustering generated 13 clusters in 7,250 SFMCs and 2,336 PBMCs shown using a UMAP projection **(A).** These clusters were manually combined into nine metaclusters corresponding to canonical immune cell lineages **(B)**. Clustering of the immune cells was performed in Seurat and immune cell metaclusters were annotated using the expression patterns of well-known cell type-specific surface proteins and genes for each metacluster. A dot plot heatmap of selected surface protein expression in the metaclusters **(C)**.

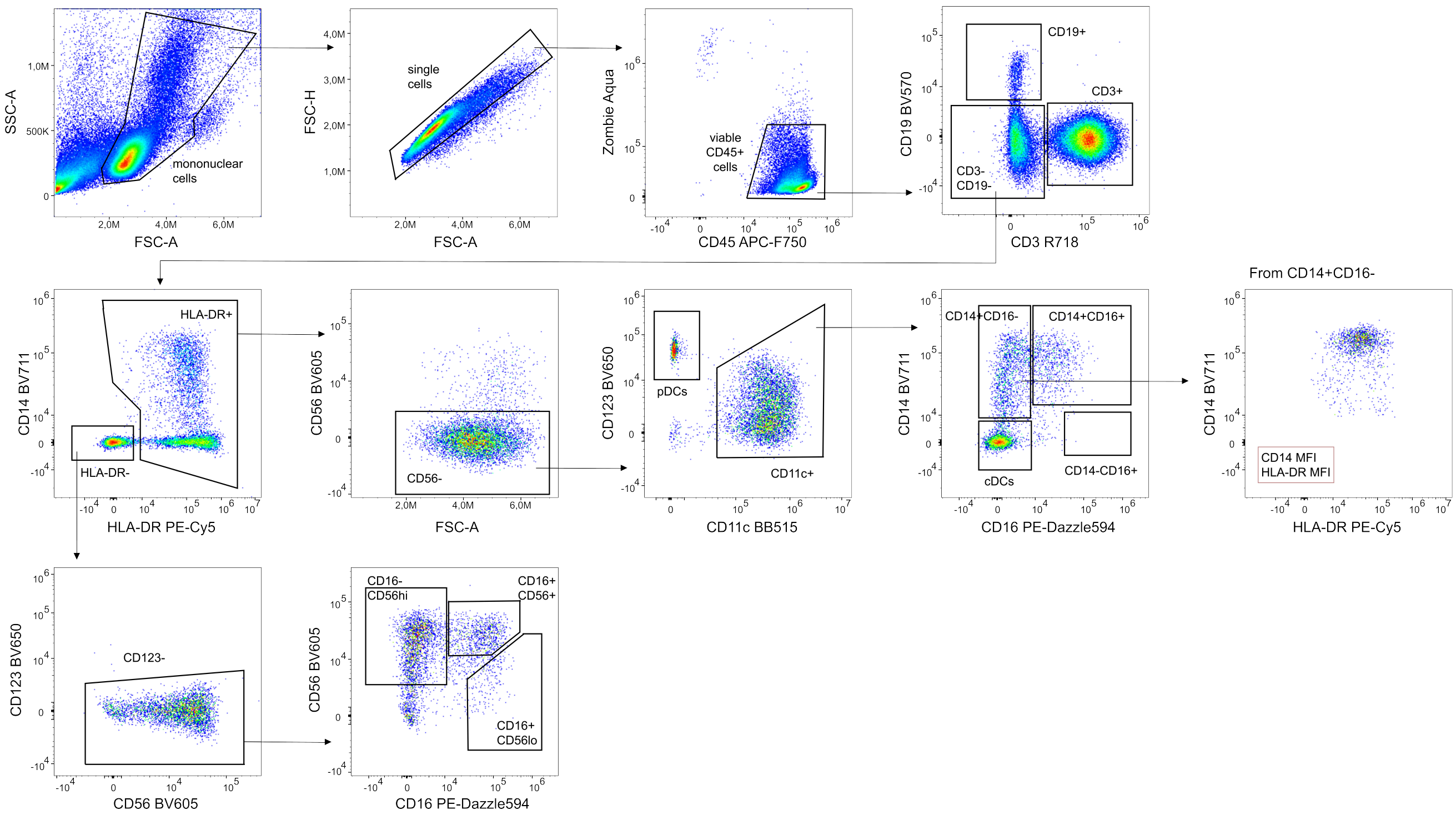

Supplemental Figure 2. Representative flow cytometry gating of monocytes/macrophages, DCs and NK cells from a JIA SFMC sample. Mononuclear cells were first gated based on FSC-A and SSC-A parameters, and singlets were determined using FSC-A and FSC-H parameters. Viable immune cells were gated as $CD45^{+}ZombieAqua^{-}$ events. pDCs, cDCs, $CD14^{+}CD16^{-}$ monocytes/macrophages, $CD14^{+}CD16^{+}$ monocytes/macrophages, $CD14^{-}CD16^{+}$ monocytes/ macrophages, as well as $CD16^{-}CD56^{hi}$, $CD16^{+}CD56^{+}$ and $CD16^{+}CD56^{lo}$ NK subsets were subsequently gated as indicated.

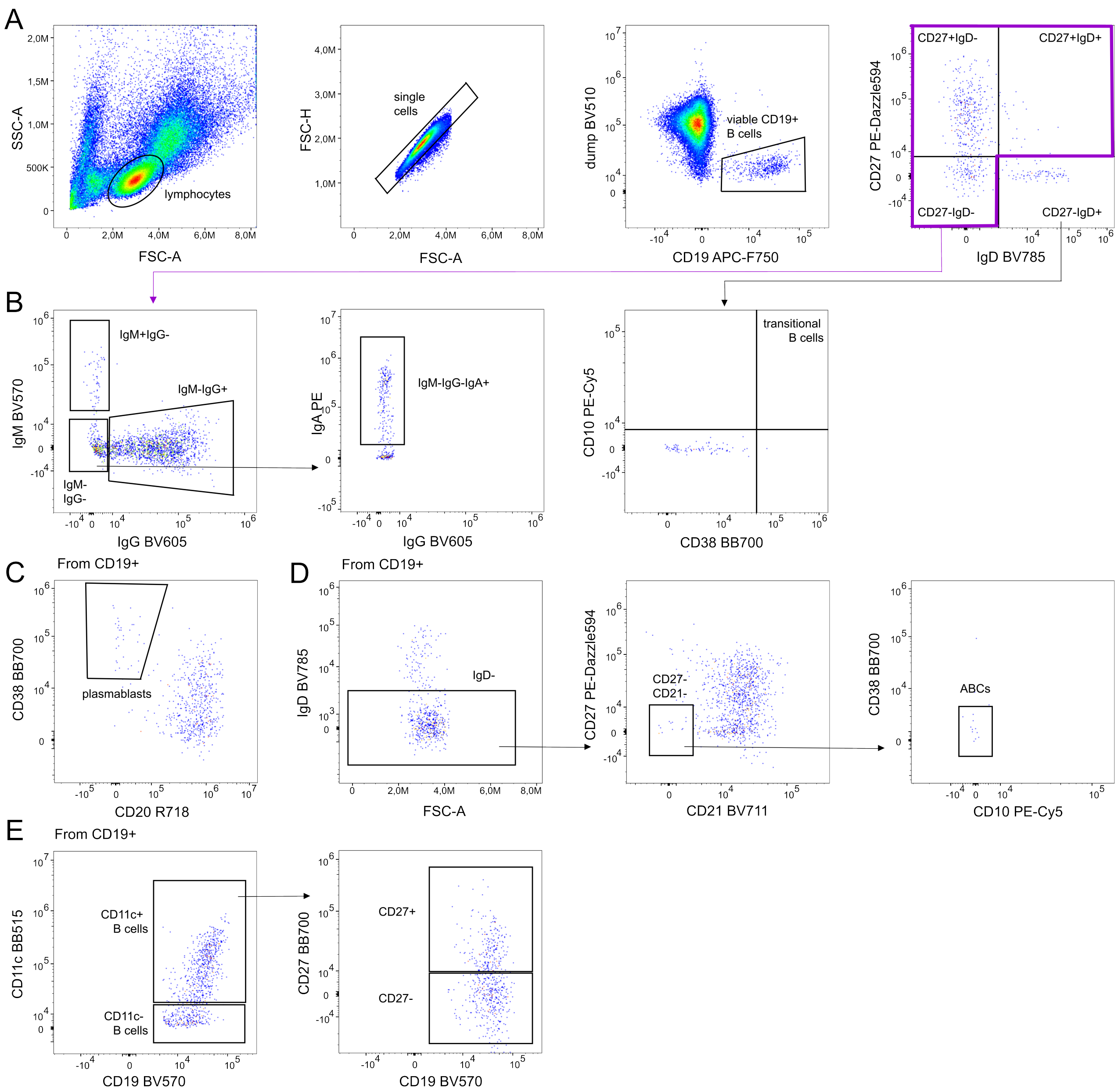

Supplemental Figure 3. Representative flow cytometry gating of B cells from a JIA SFMC sample. Lymphocytes were first gated based on FSC-A and SSC-A parameters, and singlets were determined using FSC-A and FSC-H parameters. Viable CD19$^+$ B cells were gated after exclusion of ZombieAqua$^+$, CD3$^+$, CD14$^+$, CD56$^+$ or CD16$^+$ events (dump channel), followed by gating of naïve and memory B cell subsets based on IgD and CD27 expression **(A)**. Representative gating of IgM-, IgG- and IgA-positive memory B cells and transitional B cells **(B).** Representative gating of plasmablasts **(C)** and age-associated B cells (ABCs) **(D-E).** CD11c expression was analyzed from the CD19$^+$ B cell gate shown in Supplemental Figure 2 **(E)**.

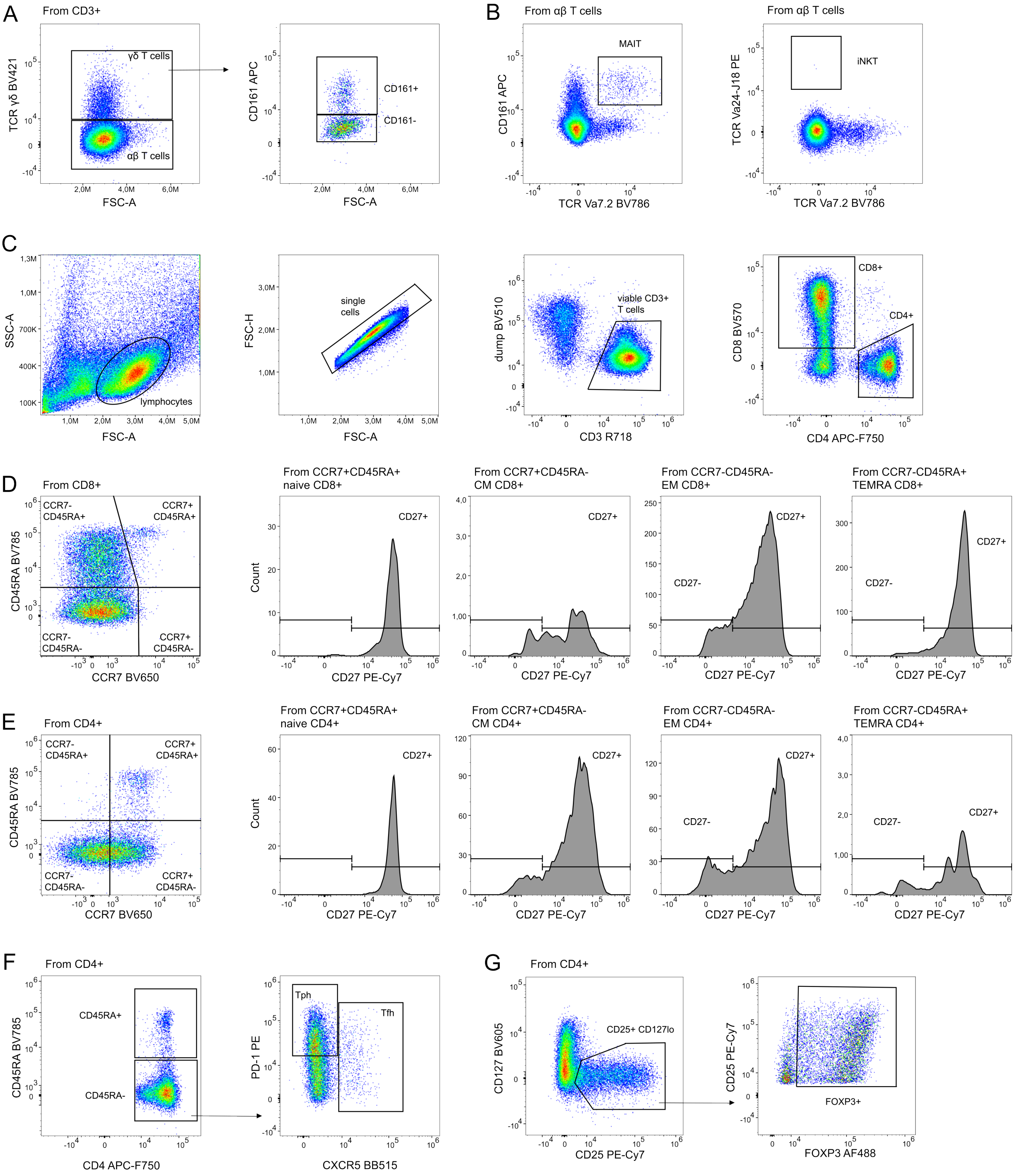

Supplemental Figure 4. Representative flow cytometry gating of T-cell subsets from a JIA SFMC sample. Unconventional T cells were analyzed from the $CD3^{+}$ T cell gate shown in Supplemental Figure 2. Representative gating of $CD161^{+/-}$ γδ T cells **(A)**, and MAIT and iNKT cells **(B)** is shown. For analysis of $CD4^{+}$ and $CD8^{+}$ T-cell subsets, lymphocytes were first gated based on FSC-A and SSC-A parameters, and singlets were determined using FSC-A and FSC-H parameters. Viable $CD3^{+}$ T cells were gated after exclusion of $ZombieAqua^{+}$, $CD19^{+}$, $CD14^{+}$, $CD56^{+}$ or $CD16^{+}$ events (dump channel), followed by gating of $CD8^{+}$ and $CD4^{+}$ T cells **(C)**. $CD8^{+}$ **(D)** and $CD4^{+}$ **(E)** naïve and memory T-cell subsets, $CXCR5^{+}$ Tfh and $CXCR5^{-}PD\text{-}1^{hi}$ Tph (**F**), and $CD25^{+}CD127^{lo}FOXP3^{+}$ Treg subsets (**G**) were subsequently gated as shown.

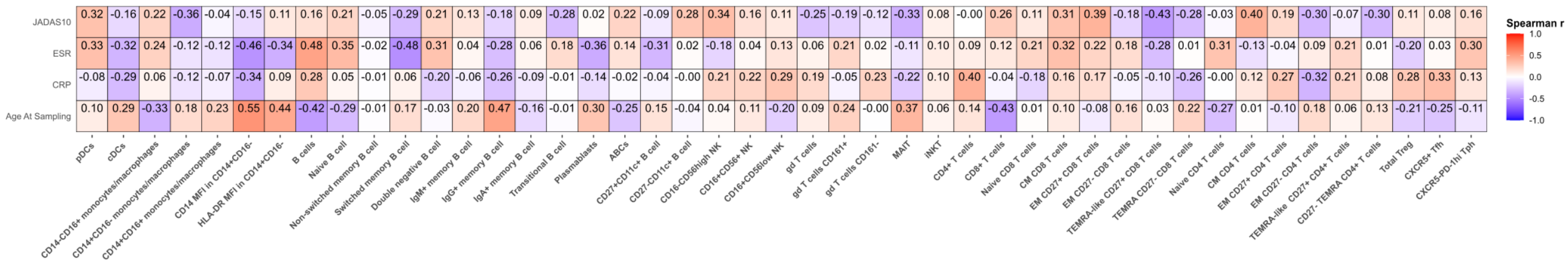

Supplemental Figure 5. Correlation between SF immune cell frequencies and continuous clinical parameters in JIA patients. Spearman's correlation was used to generate the correlation matrix between cell subset frequencies and continuous clinical variables (JADAS10, ESR, CRP, and age at sampling). Numbers within each square indicate the calculated Spearman's correlation coefficients, and the color scale represents positive correlations in shades of red and negative correlations in shades of blue. None of the correlations were statistically significant after FDR correction.

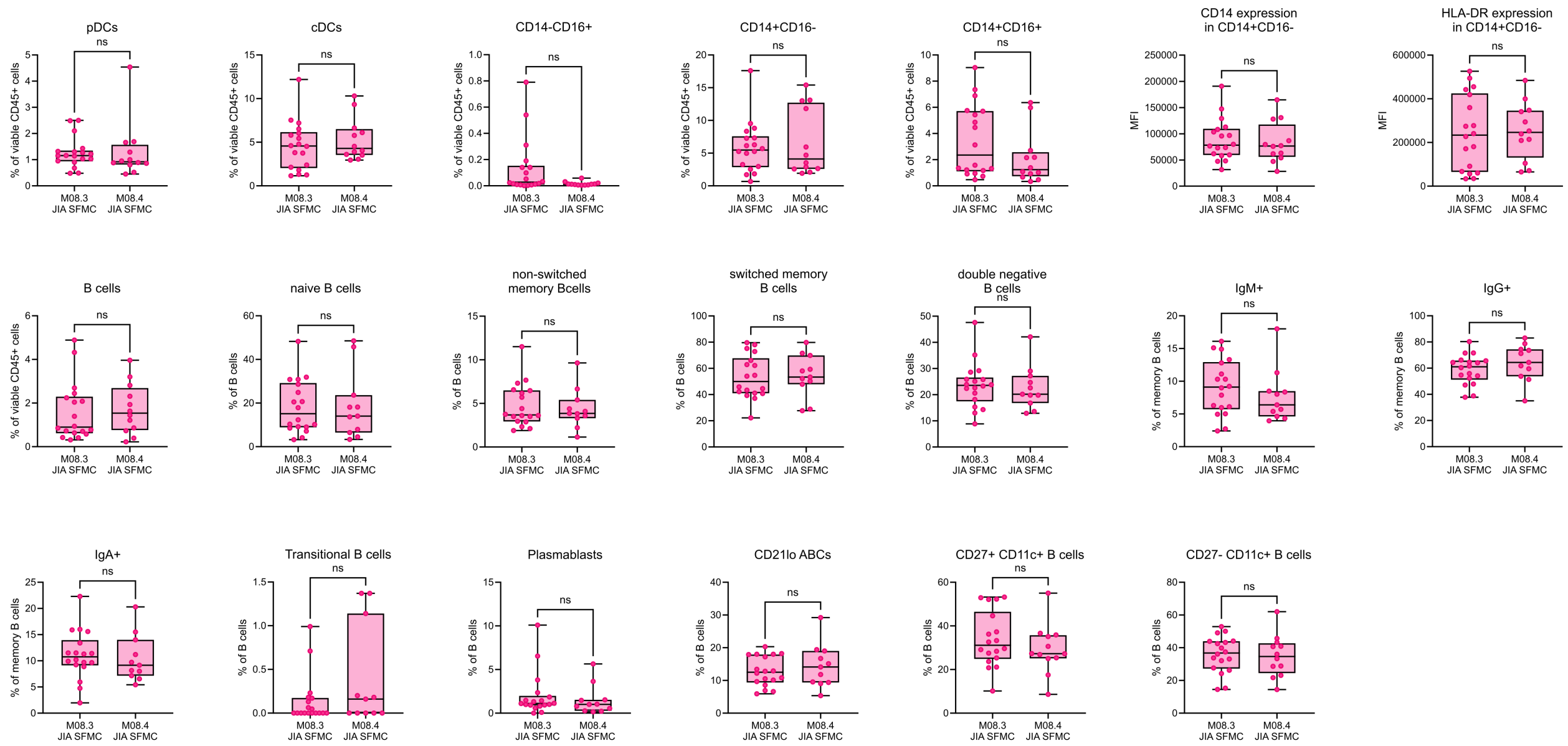

Supplemental Figure 6. Frequencies of DCs, monocyte/macrophage subsets and B-cell subsets in SF of JIA patients with the diagnosis of rheumatoid factor-negative polyarthritis (M08.3) or oligoarthritis (M08.4). Mann-Whitney U test was used for statistical testing. ns= non-significant.

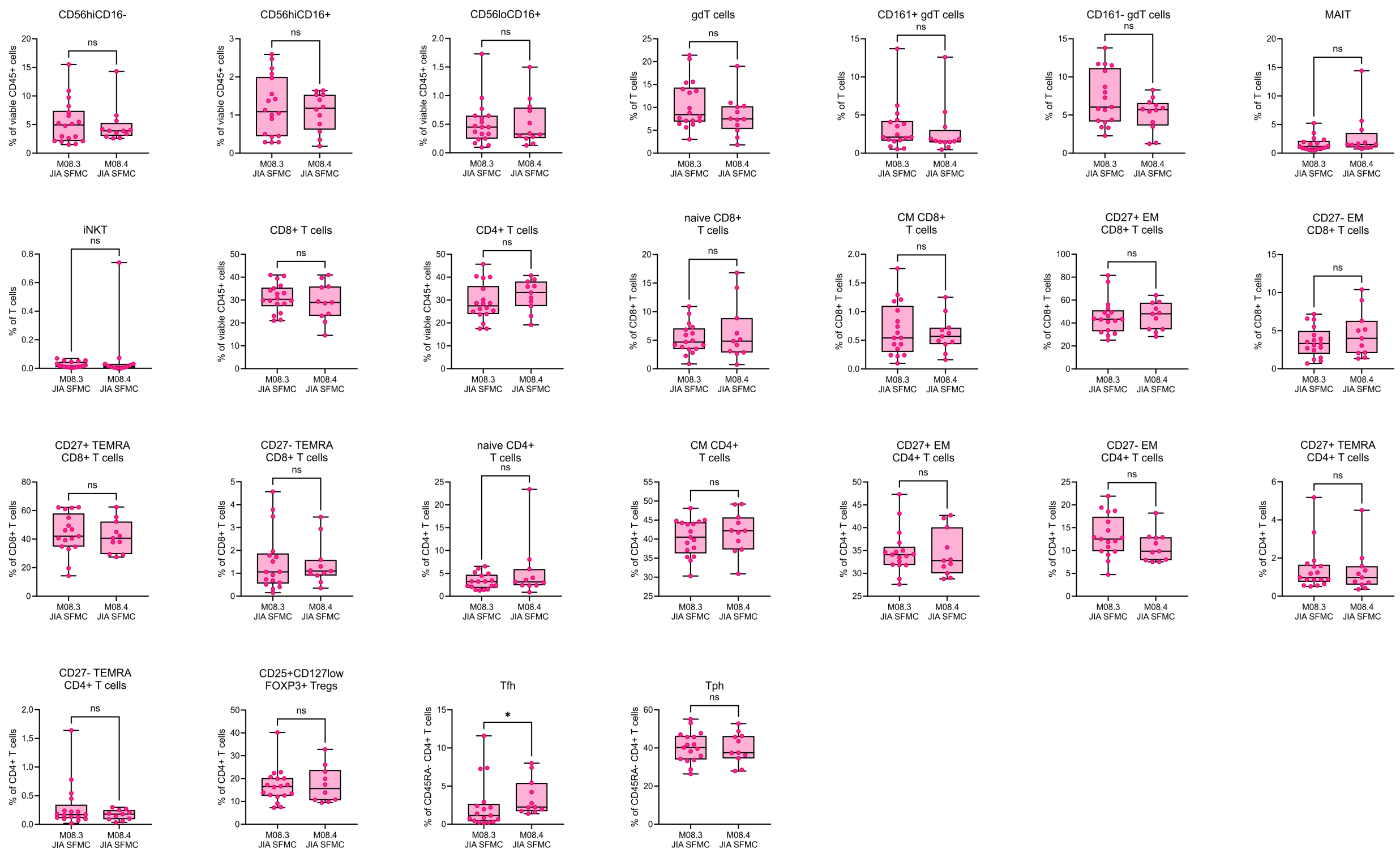

Supplemental Figure 7. Frequencies of NK and T-cell subsets in SF of JIA patients with the diagnosis of rheumatoid factor-negative polyarthritis (M08.3) or oligoarthritis (M08.4). Mann-Whitney U test was used for statistical testing. Statistical significance is indicated as $*P < 0.05$. ns= non-significant.
